# Functional analysis revealed the involvement of ZmABCB15 in resistance to *rice black-streaked dwarf virus* infection

**DOI:** 10.1186/s12870-022-03861-w

**Published:** 2022-10-11

**Authors:** Runqing Yue, Qi Sun, Jianguo Ding, Wenlan Li, Wencai Li, Meng Zhao, Shouping Lu, Tingru Zeng, Hua Zhang, Suxian Zhao, Shuanggui Tie, Zhaodong Meng

**Affiliations:** 1 Maize Research Institute, Shandong Academy of Agricultural Sciences, Jinan, China; 2 Weihai Academy of Agricultural Sciences, Weihai, China; 3 Henan Academy of Agricultural Sciences Zhengzhou, Zhengzhou, China

**Keywords:** Virus resistance, Biological control, Disease resistance, Genetics

## Abstract

**Background:**

Maize rough dwarf disease (MRDD), caused by *rice black-streaked dwarf virus* (RBSDV) belonging to the *Fijivirus* genus, seriously threatens maize production worldwide. Three susceptible varieties (Ye478, Zheng 58, and Zhengdan 958) and two resistant varieties (P138 and Chang7–2) were used in our study.

**Results:**

A set of ATP-binding cassette subfamily B (ABCB) transporter genes were screened to evaluate their possible involvements in RBSDV resistance. In the present study, *ZmABCB15*, an ABCB transporter family member, was cloned and functionally identified. Expression analysis showed that *ZmABCB15* was significantly induced in the resistant varieties, not in the susceptible varieties, suggesting its involvement in resistance to the RBSDV infection. *ZmABCB15* gene encodes a putative polar auxin transporter containing two trans-membrane domains and two P-loop nucleotide-binding domains. Transient expression analysis indicated that ZmABCB15 is a cell membrance localized protein. Over-expression of *ZmABCB15* enhanced the resistance by repressing the RBSDV replication ratio. ZmABCB15 might participate in the RBSDV resistance by affecting the homeostasis of active and inactive auxins in RBSDV infected seedlings.

**Conclusions:**

Polar auxin transport might participate in the RBSDV resistance by affecting the distribution of endogenous auxin among tissues. Our data showed the involvement of polar auxin transport in RBSDV resistance and provided novel mechanism underlying the auxin-mediated disease control technology.

**Supplementary Information:**

The online version contains supplementary material available at 10.1186/s12870-022-03861-w.

## Background

Maize rough dwarf disease (MRDD) is a major cause of crop losses in the maize (*Zea mays* L.) cultivation areas of Europe, Asia and South America [1–3]. In recent decades, MRDD has become a serious threat in the Yellow and Huai River valley, a major summer corn belt in China [4]. Previous studies have revealed three pathogens that cause MRDD, including *Maize rough dwarf virus* (MRDV), *Mal de Rio Cuarto virus* (MRCV), and *rice black-streaked dwarf virus* (RBSDV) [5]. In China, the major causal agent was designated and confirmed to be RBSDV [6].

RBSDV, a classic member of the genus *Fijivirus* in the family *Reoviridae*, is transmitted by small brown planthopper (*Laodelphax striatellus*). High density of viruliferous planthopper causes the outbreak of MRDD in the RBSDV susceptible maize varieties [7]. Application of strong insecticide to control the population of planthopper is a common method to reduce MRDD [8]. Due to high pesticide residues, chemical methods are high risk and low efficiency.

To restrain the spread of MRDD in crop fields, screening and application of intrinsic host resistance genes is one of the most cost-effective and environmentally friendly approaches [9]. In China, a number of maize cultivated varieties with different resistances to RBSDV infection have been screened and used in agriculture. However, few MRDD resistance-related genes have been identified. Recently, Rad GDP dissociation inhibitor alpha was identified as a host susceptibility factor for RBSDV resistance. βγ subunit of *Arabidopsis* SNF1 kinase (ZmAKINβγ-1) homolog in maize and ZmAKINβγ-2, two proteins with high sequence similarities, play important roles in the defense against RBSDV infection by regulating the primary carbohydrate, metabolism [10]. By comparative genomic analysis of the resistant inbred line (X178) and susceptible line (Ye478), *Zea mays Eukaryotic Initiation Factor 4E* (*ZmeIF4E*) was considered as a candidate gene for MRDD resistance [11]. In rice, several resistance genes conferring RBSDV also were identified. For examples, editing of *OseIF4G* gene confers partial resistance of rice seedlings to RBSDV [12]. A rice LRR receptor-like protein and its adaptor kinase SOBIR1 were reported to be involved in immunity against RBSDV infection [13]. Rice ARGONAUTE (AGO) proteins play crucial roles in plant defense against RBSDV invasion by regulating *HEXOKINASE 1* expression [14].

Auxin plays a role in triggering resistance to pathogen infection [15]. Several rice *Auxin Response Factor (ARF)* genes, such as *OsARF11*, *12* and *16*, involved in antiviral immune responses against rice dwarf virus invasion [16]. Rice *Auxin/indole-3-acetic Acid* (*Aux/IAA*) *10* is a target gene of rice dwarf virus P2 protein during viral infection and disease development [17]. *Tobacco mosaic* virus directs the reprogramming of the Aux/IAA gene expression in the vascular phloem to enhance virus phloem loading and systemic spread [18, 19]. Auxin participates in tomato spotted wilt virus resistance by regulating the expression of the *ARF8-miRNA167a* module [20]. In maize, Auxin Binding Protein 1 reinforces the resistance of seedlings to sugarcane mosaic virus [21].

The roles of auxin in temporal regulation of resistance to pathogen infections have been well-studied [22]. A number of auxin transport proteins are responsible for auxin polar transport from apical meristems down towards roots and other organs [23]. The auxin transport system consists of three major families, including Auxin Resistant (AUX) 1/like AUX1 influx carriers, PIN-formed efflux carriers and ATP-binding cassette (ABC) transporters [24–26]. Despite an expanded family of ABC transporters, only a few ABC orthologs have been extensively characterized in maize [27].

Some ABCB transporters are induced by auxin treatment, and their loss-of-function mutants display various auxin imbalance-related phenotypes [28]. In *Arabidopsis*, more than twenty full-size ABCB genes have been identified and at least *ABCB1*, *ABCB4*, *ABCB14*, *ABCB19* and *ABCB21* are solidly involved in auxin transport [29]. *Arabidopsis* mutants *abcb1* and *abcb19*, as well as their double mutant *abcb1*/*abcb19*, showed overlapping dwarf phenotypes and similar drastic reductions in polar auxin transport [30]. AtABCB4 functions primarily in the transport of auxin away from the root tip into the root epidermis [31]. AtABCB21 regulates the endogenous auxin levels and controls the development of cotyledons, roots and leaves [32]. Our previous study identified 65 maize auxin transporters, including 35 ZmABCB family members. The expression analysis showed that most of the ZmABCB genes were involved in responses to abiotic stresses, such as salt and drought, and cold stresses [25]. How ZmABCB family genes respond to biotic stresses, especially virus infection, was investigated in the present study. In the present study, *ZmABCB15* was cloned and its function was identified. Over-expression of *ZmABCB15* gene has positive effect on the inhibition of viral replication in maize seedlings. Our data showed the involvement of auxin in the resistance to RBSDV infection and uncovered the novel mechanism underlying auxin-mediated disease control technology.

## Results

### Evaluation of rough dwarf disease resistance of various maize varieties

**Fig. 1:**
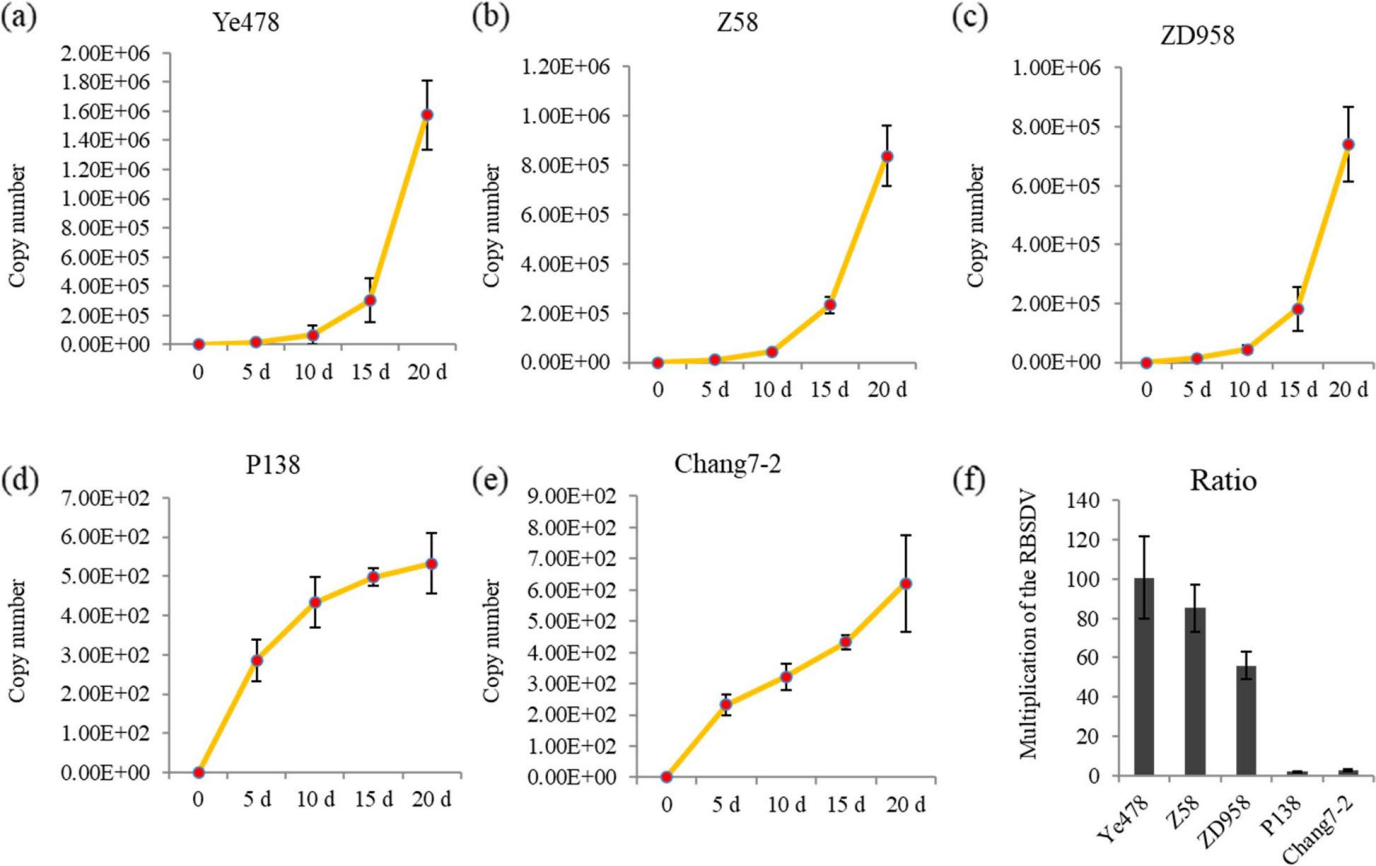
Comparison of viral titer after removal of the viruliferous planthopper in different maize varieties. The copy numbers of RBSDV in three susceptible varieties, including Ye478 (**a**), Z58 (**b**), and ZD958 (**c**), and in two resistant varieties, including P138 (**d**) and Chang7–2 (**e**). **f** Multiplication of the RBSDV in five selected varieties

The copy numbers of RBSDV were detected in leaves of all five maize varieties after inoculation with viruliferous planthoppers at different time points. The viral titers and replication rates of RBSDV in Ye478, Z58, and ZD958 are greater than P138 and Chang7–2 (Fig. 1a-e). Multiplications of the RBSDV at 20 d after removal of the viruliferous planthopper in Ye478, Z58, and ZD958 were 100.6, 85.2, and 55.9 folds higher than that at 5 d, respectively. Multiplications of the RBSDV in P138 (1.9 folds) and Chang7–2 (2.7 folds) were greatly inhibited at very a low level (Fig. 1f). The disease rate of the five varieties is provided in Additional file 1. The results confirmed that Ye478, Z58, and ZD958 are susceptible varieties and P138 and Chang7–2 are resistant varieties.

### Expression analysis of the ZmABCB family in various maize varieties

**Fig. 2:**
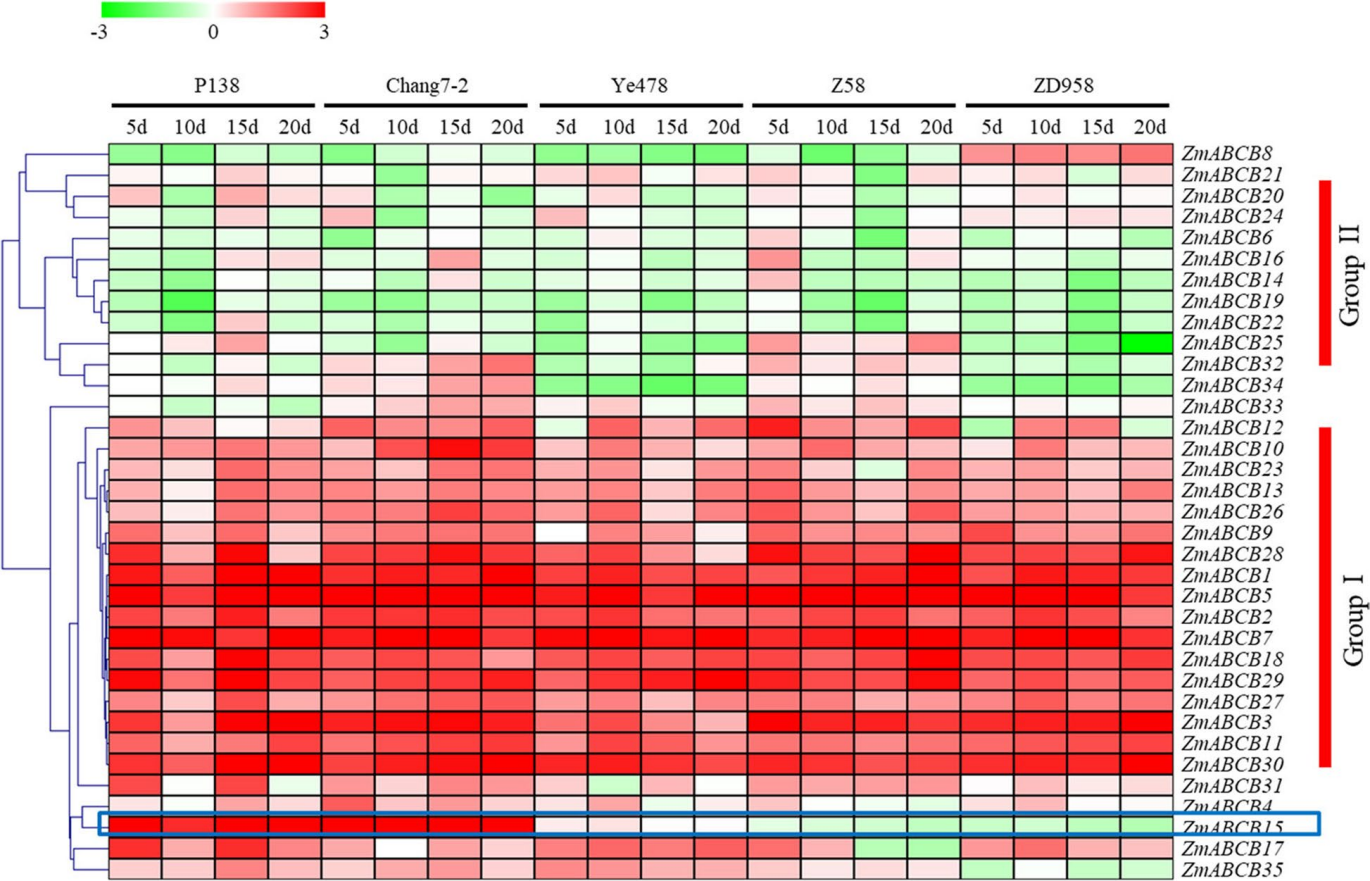
Expression analysis of the ABCB family genes under RBSDV infection. Five maize varieties, including three susceptible varieties (Ye478, Z58 and ZD958) and in two resistant varieties (P138 and Chang7–2). Compared with the control, four time points, 5d, 10d, 15d, and 20d, were selected to analyze the expression profiles of ZmABCB family. Expression profiles of maize ABCB family genes showed by a heatmap. Red indicated up-regulated and green indicated down-regulated during RBSDV infection. The heatmap scale ranges from − 3 to + 3 on a log2 scale

In order to investigate the potential roles of ZmABCB family members in response to RBSDV infection, the expression patterns of 35 ZmABCB genes in the leaves of five selected varieties were compared. Our data showed that several ZmABCB genes were significantly up-regulated by the RBSDV infection in all five selected varieties (Group I). Some other ZmABCB genes were slightly down-regulated or kept stable in all five selected varieties (Group II) (Fig. 2). Interestingly, the expression level of *ZmABCB15* was significantly induced in the resistant varieties and sightly down-regulated in the susceptible varieties. The expression level of *ZmABCB15* gene is positively correlated with the resistance to MRDD among the five maize lines, suggesting its involvement in the regulation of resistance to the RBSDV infection.

### Cloning and structural analysis of ZmABCB15

**Fig. 3:**
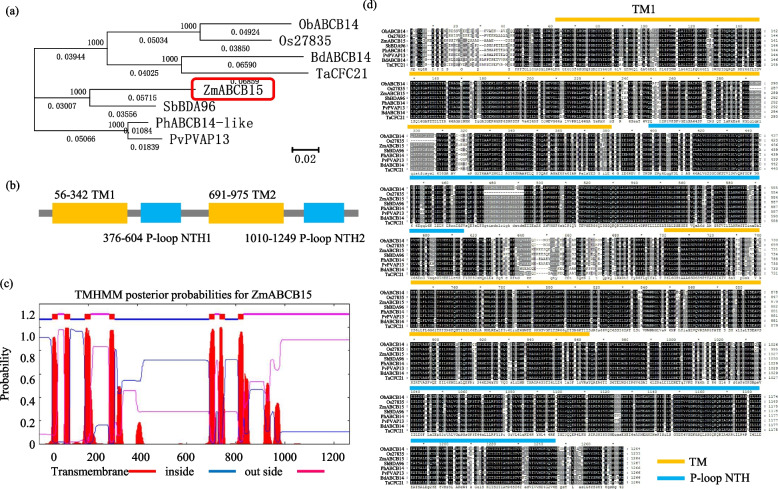
Basic biological analysis of ZmABCB15. **a** Evolutionary tree analysis of ZmABCB15 with other ABCB family members from different plants. Red box indicated the position of ZmABCB15. **b** Protein structure analysis of ZmABCB15. Yellow boxes indicated trans-membrane domains (TMs) and blue boxes indicated P-loop NTH domains. Numbers indicated the position of each domain in the ZmABCB15 protein. **c** Trans-membrane structure analysis of ZmABCB15 protein. Red lines indicated “transmembrane” structure, blue lines indicated “inside” structure, and purple lines indicated “outside” structure. **d** Multiple sequence alignment of ZmABCB15 and other ABCB members from different plants. Yellow lines indicated TM, and blue lines indicated P-loop NTH domains

Based on the genome of maize, the full-length sequence of *ZmABCB15* was cloned using PCR amplification. Sequence analysis showed that *ZmABCB15* encodes a putative transporter of 1197 amino acid residues with a calculated molecular weight of 130.75 kDa and a predicated isoelectric point of 5.80. Blast hits and phylogenetic analysis indicated significant similarity between ZmABCB15 and other ABCB family members from different plants, especially the sorghum protein SbBDA96 (Fig. 3a). Protein domain analysis showed two trans-membrane domains (TMs, locations in 56–342 and 691–9759, respectively) and two P-loop nucleotide-binding domains (NBDs, locations in 376–604 and 1010–1249, respectively) (Fig. 3b). The two TM domains were separated by a less conserved linker loop (Fig. 3c). Multiple sequence alignment revealed that ZmABCB15 protein was a classic ABCB family member, containing a universal structure similar to other ABCB family members from different plants (Fig. 3d).

### Basic biological function analysis of ZmABCB15

**Fig. 4:**
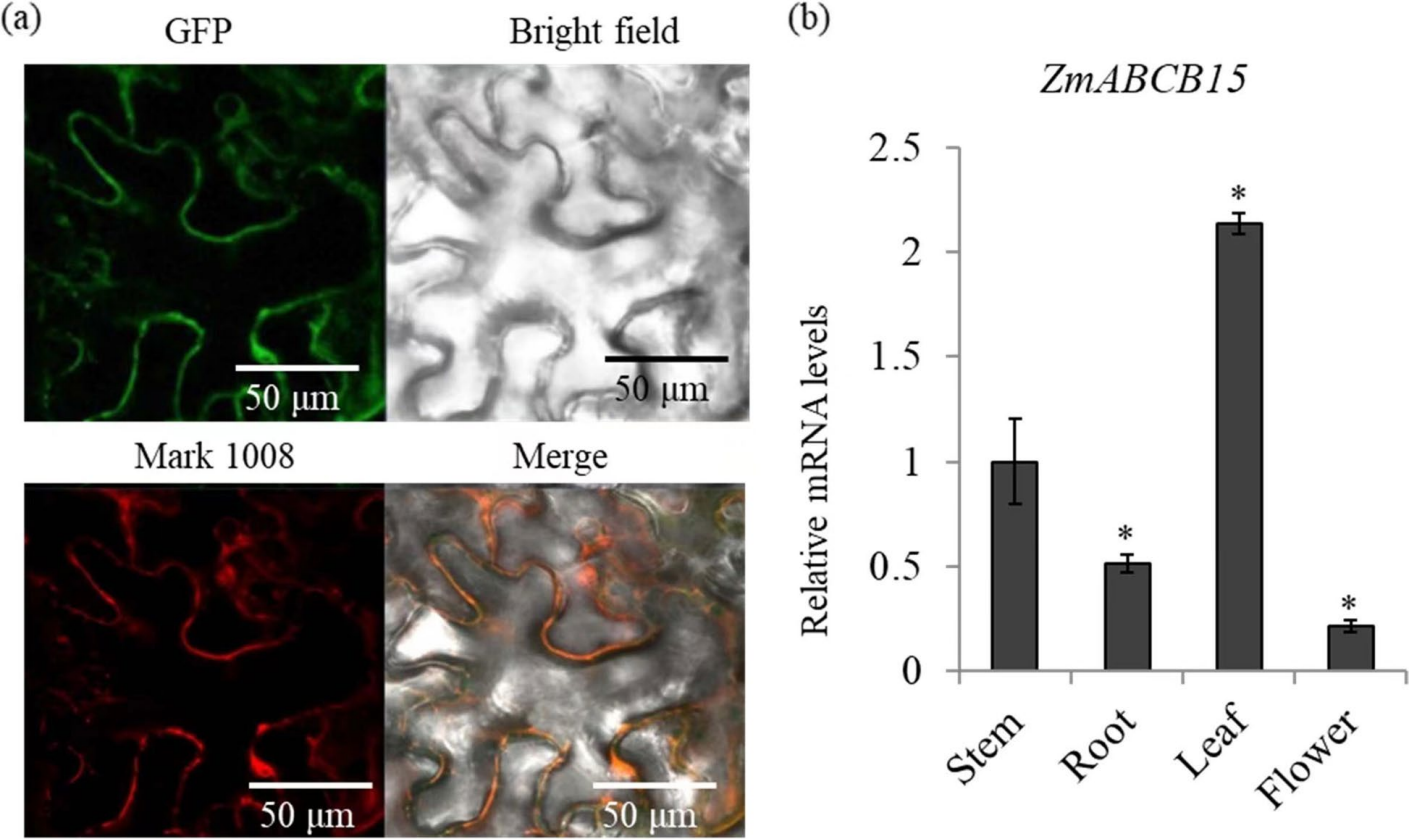
Basic biological function analysis of ZmABCB15. **a** Subcellular localization of ZmABCB15 were transiently expressed in tobacco epidermis cells. Co-localization of ZmABCB15-GFP fusion protein with the plasma membrane marker pm-rb CD3–1008, a fusion protein of a red fluorescent protein with a plasma membrane-localized aquaporin. **b** Tissues-specific expression pattern of *ZmABCB15.* The expression of ZmABCB15 in four organs, including stem, root, leaf, and flower

To determine the subcellular localization of ZmABCB15, bioinformatics analysis was performed. WoLFPSORT prediction showed that ZmABCB is an integral membrane protein. To conformed the subcellular localization of ZmABCB15, Enhanced Green Fluorescent Protein (eGFP)-ZmABCB15 fused protein was transiently expressed in tobacco epidermal cells. Our data showed that ZmABCB15 was localized to the cell membrane, consistent with the prediction that ZmABCB15 functions as an auxin transporter (Fig. 4a). In order to investigate the potential biological function of *ZmABCB15* gene, the tissues-specific expression pattern of *ZmABCB15* was analyzed. *ZmABCB15* showed the highest expression level in the stems and the lowest expression level in the flowers (Fig. 4b).

### Over-expression of *ZmABCB15* in RBSDV susceptible variety

**Fig. 5:**
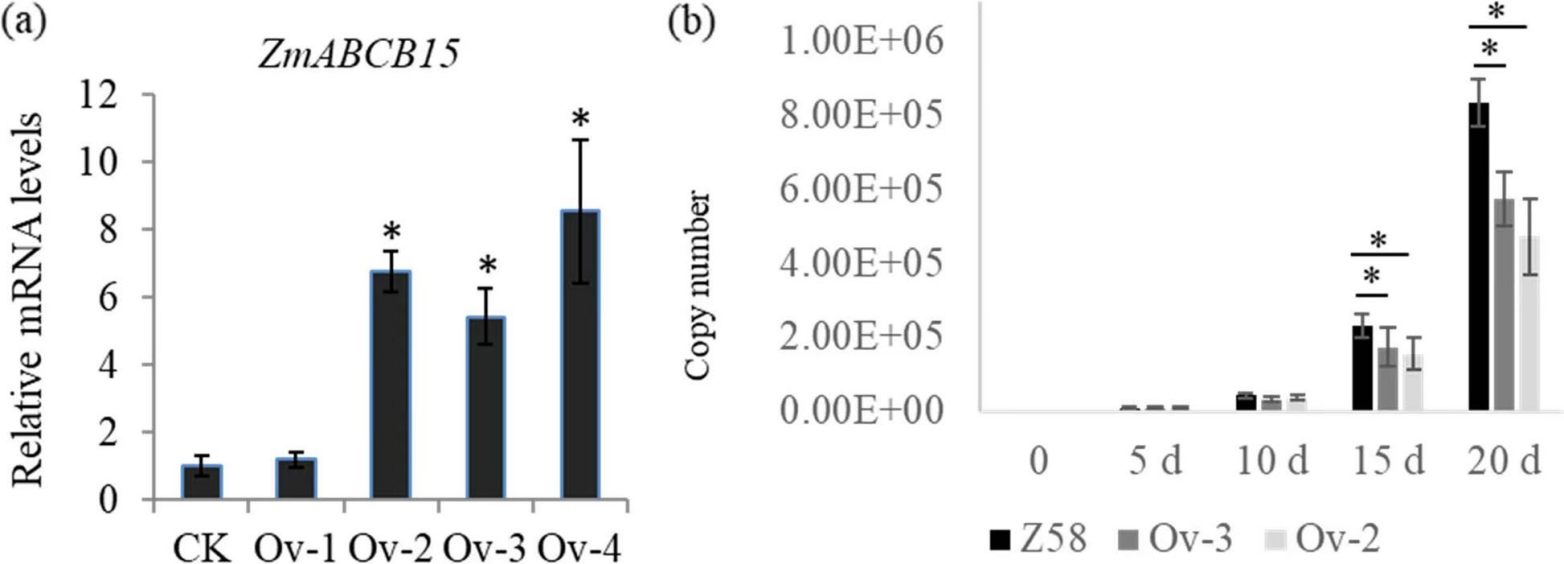
Over-expression of ZmABCB15 in RBSDV susceptible variety. **a** The expression level of *ZmABCB15* gene in different over-expression lines. Values are means ± SD of five independent replicates. Significant differences in CK and over-expression lines are indicated by “*”, *P* < 0.05. **b** Comparison of viral titer after removal of the viruliferous planthopper in Z58, Ov-3, and Ov-4

To analyze and confirm the contribution of ZmABCB15 to RBSDV resistance in maize, we used the *Agrobacterium*-mediated transformation technique to produce *ZmABCB15* over-expression lines. Four transgenic lines of T1 generation were used to determine the copy number and expression level of *ZmABCB15* gene. Our data showed that the Ov-1 and Ov-4 lines contained two insert copies, and Ov-2 and Ov-3 contained one insert copy. The relative expression level of *ZmABCB15* gene was significantly up-regulated in Ov-2, Ov-3 and Ov-4 lines, and not in the Ov-1 line (Fig. 5a). Single copy lines are conducive to screening genetically stable transgenic homozygous plants and accelerating the process of gene biological breeding. Thus, the Ov-2 and Ov-3 lines were used to test disease resistance to RBSDV. No obvious phenotypic differences were observed between wide type (WT) and ZmABCB15 overexpression lines. The phenotype of WT, Ov-2, and Ov-3 under RBSDV infection is provided as Additional file 2.

### ZmABCB15 repressed the RBSDV replication ratio

To confirm the role of *ZmABCB15* in RBSDV resistance, the RBSDV replication ratio was determined in the control and *ZmABCB15* over-expressed lines. RBSDV was detected in leaves of the control and *ZmABCB15*-Ov lines after inoculation with viruliferous planthoppers. Compared with the control, the titers of virus in Ov-2 and Ov-3 lines were smaller and increased slightly after inoculation (Fig. 5b). Taking time point 5 d as control, the viral titer at 20 d increased 85 folds in the control seedlings. Taking time point 5 d as control, the viral titer at 20 d increased 68 and 69 folds at 20 d in the Ov-2 and Ov-3, respectively. Our data indicated that the viral replications in the Ov-2 and Ov-3 lines were inhibited, and Ov-2 showed stronger inhibitory effect on the viral replication than Ov-3.

### Analysis of endogenous auxin contents

**Fig. 6:**
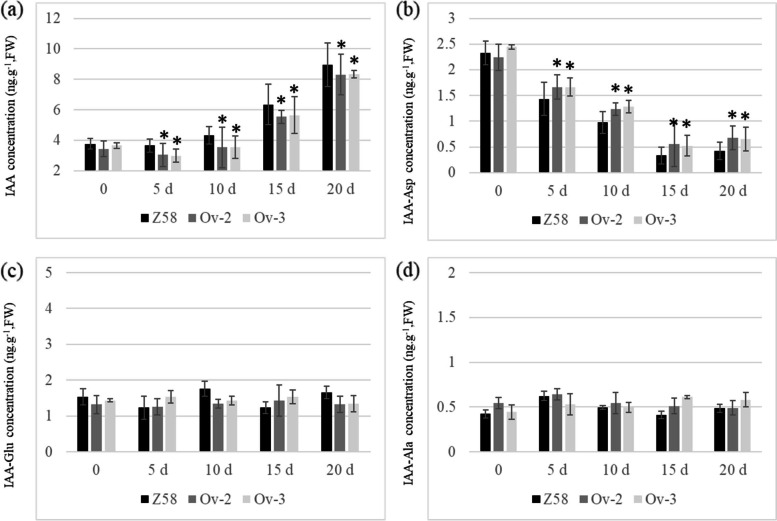
Analysis of the role of ZmABCB15 in resistance to RBSDV infection. **a** Free IAA contents in Z58, Ov-3 and Ov-4 lines. **b** IAA-Asp contents in Z58, Ov-3 and Ov-4 lines. **c** IAA-Glu contents in Z58, Ov-3 and Ov-4 lines. **d** IAA-Ala contents in in Z58, Ov-3 and Ov-4 lines. Values are means ± SD of five independent replicates. Significant differences in Z58 and two over-expression lines are indicated by “*”, *P* < 0.05

To analyze how RBSDV infection affects endogenous auxin transport, the 3-Indoleacetic acid (IAA) contents in control Z58 and *ZmABCB15* over-expressing lines were determined. Compared with the un-infected seedlings (0 d), RBSDV infection significantly induced the free IAA contents and reduced the IAA-Asp contents in all tested maize lines, including Z58, Ov-2 and Ov-3, at the same time points (Fig. 6a and b). However, the free IAA and IAA-Asp change rates in Ov-2 and Ov-3 lines were obviously lower than that in Z58 (Fig. 6a and b). At the time points 10 d, 15 d, and 20 d, the free IAA concentration in Z58 were significantly higher than that in Ov-2 and Ov-3. Compared with the un-infected seedlings (0 d), the IAA-Glu and IAA-Ala contents remained constant after RBSDV infection (Fig. 6c and d).

### Role of auxin polar transport inhibitor in the IAA contents

**Fig. 7:**
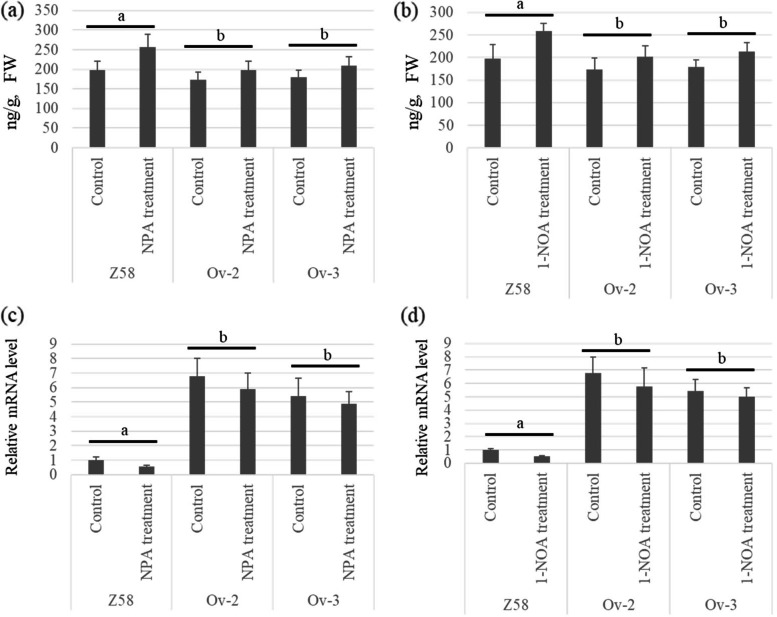
Role of auxin polar transport inhibitor in the IAA content and ZmABCB15 expression. **a** The effect of NPA on IAA contents in Z58, Ov-2, and Ov-3 lines. **b** The effect of 1-NOA on IAA contents in Z58, Ov-2, and Ov-3 lines. **c** The effect of NPA on *ZmACBC15* expression in Z58, Ov-2, and Ov-3 lines. **d** The effect of 1-NOA on *ZmACBC15* expression in Z58, Ov-2, and Ov-3 lines

To reveal the role of polar auxin transport in the changes of endogenous IAA contents, two auxin polar transport inhibitors, N-1-naphthylphthalamic acid (NPA) and 1-naphthoxyacetic acid (1-NOA), were used to block auxin transport. Our data showed that application of NPA significantly increased the endogenous IAA contents in the leaves of both *ZmABCB15* over-expression lines (Ov-2 and Ov-3) and control Z58. In detail, the IAA contents in leaves of Z58 was 197.18 ng/g, and in the leaves of Ov-2 and Ov-3 were 173.29 ng/g and 179.06 ng/g, respectively. After 7 days of NPA treatment, the content of IAA in leaves of Z58 was increased from 197.18 ng/g to 257.40 ng/g, with an increase range of 30.54%. After 7 days of NPA treatment, the IAA contents in young leaves of Ov-2 and Ov-3 were increased by 14.32 and 16.50% respectively (Fig. 7a). 1-NOA application showed a similar effect to NPA treatment on both of Z58 and *ZmABCB15* over-expression lines (Fig. 7b).

### Role of auxin polar transport inhibitor in the expression of *ZmABCB15*

To reveal the role of auxin transport in the regulation of *ZmABCB15* expression, two auxin polar transport inhibitors, NPA and 1-NOA, were used to block auxin transport. After 7 days NPA treatment, the expression level of *ZmABCB15* gene was significantly down-regulated in the leaves of Z58 and only was slightly down-regulated in the Ov-2 and Ov-3 lines. In detail, the expression level of *ZmABCB15* was decreased by 45.6% in Z58 and only was deceased by 12.7 and 10.2% in Ov-2 and Ov-3, respectively (Fig. 7c). 1-NOA application showed a similar effect to NPA treatment on both of Z58 and *ZmABCB15* over-expression lines (Fig. 7d).

## Discussion

RBSDV causes several viral diseases in maize worldwide, leading to significant losses in yield of susceptible cultivars. Auxin controls many aspects of disease symptoms, particular in virus-induced abnormal growth and development [33]. In model plants, interactions between virus replicase and Aux/IAA proteins lead to alteration in Aux/IAA localization that is required for the development of disease localization [19]. However, the molecular basis of auxin-related resistance to virus infection remains poorly understood.

In maize, MRCV infection causes auxin imbalance by altering the expression of several auxin transporter encoding genes, PIN-FORMEDs (PINs) and PIN-likes [34]. In all tested maize varieties, many ZmABCB family genes, such as *ZmABCB28*, *ZmABCB1*, *ZmABCB5*, *ZmABCB2*, *ZmABCB7*, *ZmABCB18*, *ZmABCB29*, *ZmABCB27*, *ZmABCB3*, *ZmABCB11*, and *ZmABCB30*, were significantly up-regulated, suggesting an activated auxin transport in RBSDV infected seedlings.

Comparing resistant and susceptible materials is an efficient method to screen MRDD resistance genes. A large number of maize materials, including several resistant lines, such as ‘80007’, ‘K36’, ‘Mo17’, ‘Zheng58’, and ‘Zhengdan958’, and several susceptible lines, such as ‘80044’, ‘S221’, ‘Ye478’, ‘XH6’, ‘P138’, ‘Chang7–2’, and ‘Dan 340, were reported by different groups, providing abundant genetic materials for screening MRDD resistance genes [5, 35, 36]. Interestingly, a resistant varieties-specific inducted auxin transporter encoding gene, *ZmABCB15*, was identified, suggesting a potential RBSDV resistance gene.

ABCB family transporters function in long distance auxin transport and non-polar auxin distribution in vegetative organs [37]. Homologs of ABCB family genes have been reported in both monocots and dicots. In maize, loss-of-function mutations in *ZmABCB1* result in short stature plant designated as *brachytic2* [38]. Here, *ZmABCB15* was identified and cloned, aiding to understand auxin transport in monocots. In model plants, several auxin transport-related ABCB genes were reported to process a tissue specific expression pattern. For examples, *AtABCB1* is expressed in the stele tissues of the root apical region, *AtABCB4* is expressed in the root epidermis, *AtABCB14* and *AtABCB15* are expressed in the vascular tissues of the inflorescence stem, and *AtABCB19* is expressed in the hypocotyl and root [29, 39, 40]. Expression analysis showed that *ZmABCB15* was primarily expressed in the stem, suggesting a potential role in mediating root ward auxin transport.

The auxin signaling pathway consists of different components, such as TIR1/AFB proteins, Aux/IAA proteins, ARFs, and auxin transporters [41]. Several important components of the auxin signaling pathway play significant roles in the resistance of plants to virus infection [16]. Gain-of-function mutant of OsIAA10, proteins at the nexus of the auxin signaling, enhances the rice dwarf virus infection and disease development [17]. OsARF17, a member of auxin response factor family, confers the resistance to a cytorhabdovirus by directly binding to the viral proteins [42]. However, the role of auxin transporters in the resistance to viral infection is largely unknown.

RBSDV is displayed by viral replication curve, which is an important indicator of screening resistance genes [5]. Over-expression of *ZmABCB15* significantly repressed the RBSDV replication ratio, suggesting a role of auxin transporter in the resistance to RBSDV infection. In the present study, we firstly reported an auxin transporter, *ZmABCB15*, and its role in the resistance to RBSDV infection.

The abnormal symptoms of host plants suffered bacterial and fungal diseases are related to auxin imbalance. After RBSDV infection, many physiological and biochemical changes and metabolic processes in plants affect the expression of auxin transport genes and the synthesis of endogenous hormones, resulting in dwarfing, deformity, chlorosis, shrinkage and swelling of leaves. Understanding the correlation between the expression level of auxin transporter encoding genes and the changes of hormone level in maize under RBSDV infection can guide the application of transgenic technology or external hormone in the regulation and biological control of maize rough dwarf disease.

Under normal conditions, the contents of free IAA in *ZmABCB15* over-expression lines were similar to the control Z58. Addition to free IAA, the major active form of endogenous auxin, inactive IAA-Ala and two types of intermediates, including IAA-Asp and IAA-Glu, exist in plants extensively [43]. Interestingly, over-expression of *ZmABCB15* alleviated the increasing of free IAA and enhanced the rising of IAA-Asp in transgenic plants compared with Z58, indicating a role of ZmABCB15 in homeostasis of active and inactive auxins in the RBSDV infected seedlings.

Our current understanding of how auxin moves between different tissues is largely built on the application of polar auxin transporter inhibitors [44]. The ability of NPA to disrupt tropic growth, but not plant growth, prompted its use as an inhibitor of polar auxin transport [45]. After NPA treatment, the auxin content was largely induced in the control Z58. The gradient distribution of auxin concentration was disordered, which affected the normal development of maize seedlings with rough dwarf disease. Auxin transporter inhibitors might affect the activity of ABCB family transporters [46]. Polar auxin transport might participate in the MRDD resistance by affecting the distribution of endogenous auxin among tissues.

## Conclusions

Our findings demonstrated that *ZmABCB15* encoding an auxin transporter involved in resistance to RBSDV infection. Expression analysis showed that *ZmABCB15* primarily expressed in the leaves and was significantly induced by RBSDV infection in the resistant varieties. Over-expression of *ZmABCB15* in RBSDV susceptible variety enhanced resistance to MRDD by repressing the RBSDV replication ratio. Over-expression of *ZmABCB15* might affect the homeostasis of active and inactive auxins in the RBSDV infected seedlings. Application of auxin polar transport inhibitor affect the IAA content and ZmABCB15 expression. Our data showed the involvement of ZmABCB15 in the resistance to MRDD and provided novel mechanism underlying the auxin-mediated disease control technology.

## Methods

### Plant and planthopper materials

Five maize varieties, including four inbred lines (Zheng58 (Z58), Chang7–2, P138, and Ye478) and one hybrid (Zhengdan 958 (ZD958)), were used in our study. Ye478, Z58 and ZD958 are susceptible varieties and P138 and Chang7–2 are resistant varieties [5, 31]. Planthoppers (*Laodelphax striatellus*) were fed on wheat cv. Zhoumai 19 seedlings in a pot covered with 80-mesh mothproof net in a greenhouse. The growth condition was set with a photoperiod of 16 h light/8 h dark, relative humidity of 60%, light intensity of 120 μmol.m^− 2^. s^− 1^, and temperature at 25°C.

### Virus transmission and leaf sampling

The virus-free small brown planthopper were fed on MRDD plants in Jinan for 3 d to acquire the virus. Then, the planthoppers with RBSDV were fed on two-week-old wheat seedlings for another a week at 25°C. Before inoculation, 50 planthoppers were randomly selected for PCR to evaluate to the RBSDV carrier rate. Three biological repeats (50 planthoppers each repeat) were performed. The RNA of each planthopper was extracted and amplified using two RBSDV specific primers (RP: 5′-GCTCCTACTGAGTTGCCTGTC-3′; RBSDV-R: 5′-TCAGCAAAAGGTAAGGAACG-3′). Total RNAs from a single planthopper were isolated using the ZYMO RESEARCH Direct-zol RNA MiniPrep Kit (Irvine, CA, USA) according to its method. Three technical repeats were performed in the PCR experiment.

Uninfected two-leaf-stage maize seedlings were inoculated with viruliferous planthoppers for 3 d. Each maize seedling was inoculated with 10 viruliferous planthoppers. RBSDV-free small brown planthopper inoculated maize seedlings were used as controls. After inoculation, the infected maize seedlings were sprayed with 5% 1-(6-chlorine-3 pyridine methyl)-N-nitroalkane-2-amino to kill the planthoppers. Then, five fully expanded leaves of infected maize seedling were collected on 0 d, 5 d, 10 d, 15 d, and 20 d after the removal of viruliferous planthoppers.

### Treatment of the maize seedings with chemicals

1-N-naphthylphthalamic acid (NPA) and 1-naphthoxyacetic acid (1-NOA), two auxin polar transport inhibitors, were purchased from Aladdin corporation (Shanghai, China). The leaves of maize seedlings were sprayed 10 mM NPA solution or 10 mM 1-NOA solution for 7 days. Deionized H_2_O was used as the control. For each treatment, three biological replicates were performed.

### RNA extraction and standard calibration curve preparation

Total RNAs from the leaves of maize seedlings were isolated using the same RNA extraction Kit. Then, 1 μg RNA was used for reverse transcription using a TAKARA D6110A kit (Kusatsu, Japan). The S7 fragment of RBSDV was amplified using two specific primers (RBSDV-F: 5′-AGAGCTCTTCTAGTTATTGCG-3′; RBSDV-R) and its 510 bp PCR product (S7 pigment of RBSDV) was treated as standard sample. The PCR product was inserted to the pMD18-T vector and its copy number was measured using a NanoDrop 2000. A 10-fold serial dilution of the S7 fragment of RBSDV was prepared. Fluorescence qRT-PCR was performed using two primers, RBSDV-R and RP. The standard calibration curve was calculated by plotting the logarithm of the copy number of serial dilutions of standard sample on the horizontal axis against the *Ct* value on the vertical axis. The method of qRT-PCR was performed according to our previous work [5]. The viral titer of maize seedlings was quantified by comparing the *Ct* values against the standard curve.

### Quantitative RT-PCR for gene expression analysis

The total RNAs were extracted from the leaves of maize seedlings using a Plant RNeasy mini-Kit (Qiagen, Hilden, Germany) according to its instruction. The internal reference gene *ZmACTIN* (LOC100284092) was used as a control to calculate the relative fold differences between different treatments by comparing the cycle threshold values (2^-ΔΔCt^). Briefly, 1 μL of cDNA in ddH_2_O was add to 5 μL of 2× SYBR, 100 nM of each primer and ddH_2_O was then added to reach final volume 10 μL. The procedures for PCR were as follows: 95 °C for 10 min; 40 cycles of 95 °C for 15 s, 60 °C for 60 s. All the expression analysis was carried out for five biological replicates and each replicate was run in triplicate. The primer sequences are listed in Additional file 3.

### Subcellular localization, phylogenetic tree building, and gene structure analysis

The full-length ZmABCB15 sequence was cloned into the pH7HWG2.0 vector to produce an artificial fused GFP protein with primers: ATGCCAGCCATCCAAGGGAA/ TTAGTCTTTGACTCCTTTGC. Two vectors, including ZmABCB15-GFP and empty-GFP, were transiently expressed in *N. benthamiana* epidermal cells by GV3101-mediated transformation. The fluorescence of the GFP fused protein was detected using a LSM710 confocal microscope (Carl Zeiss, Oberkochen, Germany).

Multiple sequence alignments were carried out using ClustalW software (http://www.clustal.org/clustal2/) with default parameters and visualized by GeneDoc software (http://www.nrbsc.org/gfx/genedoc/). An unrooted phylogenetic tree was constructed with ZmABCB15 and seven ABCB family members from other plants using MEGA6 (http://www.megasoftware.net/mega6/mega.html). The sequences of ZmABCB family are listed in Additional file 4. The transmembrane structures of ZmABCB15 were estimated using TMHMM2 online tool (www.cbs.dtu.dk/services/TMHMM/).

### Genetic transformation of ZmABCB15 in maize

For over-expression vector construction, the full-length cDNA of *ZmABCB15* from field grown B73 inbred seedlings was cloned into the pCAMBIA3301 vector with two restriction enzymes (*Hind*III and *BamH*I). Then, the construction was introduced into *Agrobacterium tumefaciens* strain EHA105 according to previously published work [32]. The ears of Z58 were collected from the seedlings 14 d after pollination and sterilized with 75% ethanol. Immature embryos were dissected from the sterilized ears and incubated with *A. tumefaciens* containing the 35S vector for 10 min. After incubation, the embryos were washed with ddH_2_O and transferred onto the resting medium, and selection medium. After 4 weeks of selection, calli with bialaphos resistance were transferred onto the subculture medium for regeneration [33].

### Southern blotting

Genomic DNA was extracted from ZmABCB15 over-expression lines of maize using cetyltrimethylammonium bromide method. Then, 10 μg of total DNAs were digested with *EcoRI*. After electrophoresis in 0.7% agarose gel, DNA samples were transferred to HyBond N^+^ membrane overnight. The sample DNA was fixed to the membrane at 80 °C for 2 h. The probes were synthesized and labeled with Digoxigenin-11-dUTP (PCR DIG Probe Synthesis Kit, Roche). DNA gel blot hybridization was performed using DIG DNA labeling and detection kit (Roche, Basel, Switzerland). The HyBond N^+^ membrane was washed three times using 2 × SSC/0.1% SDS solution for 5 min at 25 °C, then for 15 min in 1 × SSC/0.1% SDS solution at 65 °C twice. The membrane was blocked for 30 min and was immersed in Anti-Digoxigenin-AP solution for 30 min. Hybridized membrane was detected in the presence of CSPD (C_18_H_20_ClNa_2_O_7_P), and the signals were detected on X-ray film.

### Determination of endogenous auxin contents

The RBSDV infected maize seedlings and mock seedlings were grown in half-strength modified Hoagland solution. The leaf samples were harvested and washed three times with ddH_2_O. Then, the tissue samples were collected by 14, 000 g centrifugation and quickly frozen in liquid N_2_ until used. Three independent biological replicates (15 mg each sample) were purified after addition of 250 pg of ^13^C6-IAA internal standard using a ProElu C18 column (DiKMA, Beijing, China). Endogenous auxin contents were measured on FOCUS GC-DSQII system (Thermo Fisher Scientific, Austin, TX, USA).

### Statistical analysis

Student’s *t*-test analysis between mock and infected-seedlings was performed. Experiments were repeated five biological times, and standard deviations were shown with the mean of replicates±SD.

## Supplementary Information

**Additional file 1: Table S1.** The disease rates of the five varieties after inoculating RBSDV.

**Additional file 2: Fig. S1.** The phenotypes of WT, Ov-2 and Ov-3 lines. (a) The growing of WT, Ov-2 and Ov-3 lines under control condition. (b) The growing of WT, Ov-2 and Ov-3 lines under RBSDV infection conditions.

**Additional file 3: Table S2.** The CDSs of ZmABCB family.

**Additional file 4: Table S3.** The primer sequences for the qRT-PCR.

## Data Availability

All the data was submitted with the manuscript. No sequencing data was used in the present study.

## Abbreviations

ABC: ATP-binding cassette
ABCB: ATP-binding cassette subfamily B
AGO: ARGONAUTE
ARF: Auxin Response Factor
eGFP: Enhanced Green Fluorescent Protein
IAA: 3-Indoleacetic acid
MRCV: Mal de Rio Cuarto virus
MRDD: Maize rough dwarf disease
MRDV: Maize rough dwarf virus
NBD: nucleotide-binding domain
1-NOA: 1-naphthoxyacetic acid
NPA: N-1-naphthylphthalamic acid
RBSDV: rice black-streaked dwarf virus
TM: trans-membrane
PIN: PIN-FORMED
WT: wide type
ZmAKINβγ-1: βγ subunit of Arabidopsis SNF1 kinase
ZmeIF4E: *Zea mays* Eukaryotic Initiation Factor 4E
