# Supplementary material for: Functional analysis revealed the involvement of ZmABCB15 in resistance to rice black-streaked dwarf virus infection
Source: BMC Plant Biol. 2022 Oct 11;22:484. doi: 10.1186/s12870-022-03861-w (PMC9552357; doi:10.1186/s12870-022-03861-w)
Supplement: Supplementary file 2 — Additional file 2: Fig. S1. The phenotypes of WT, Ov-2 and Ov-3 lines. (a) The growing of WT, Ov-2 and Ov-3 lines under control condition. (b) The growing of WT, Ov-2 and Ov-3 lines under RBSDV infection conditions. [file 12870_2022_3861_MOESM2_ESM.pdf]

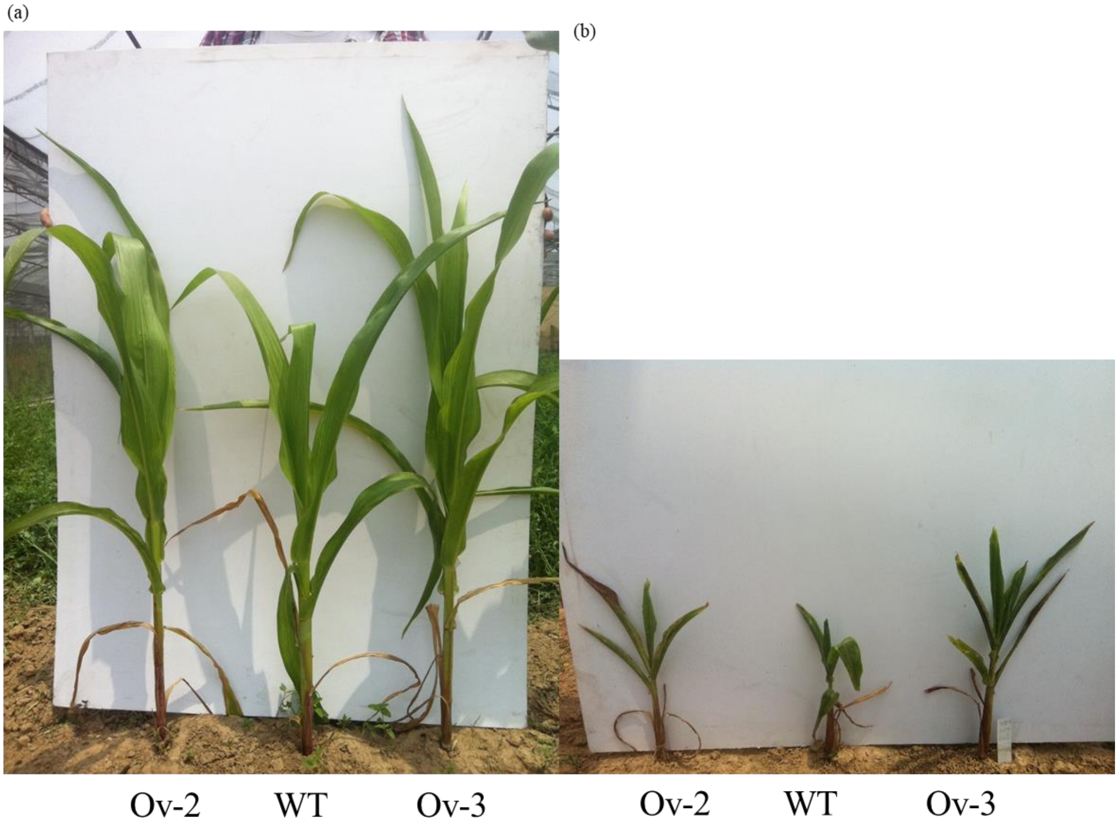

Figure S1 **The phenotypes of WT, Ov-2 and Ov-3 lines.** (a) The growing of WT, Ov-2 and Ov-3 lines under control condition. (b) The growing of WT, Ov-2 and Ov-3 lines under RBSDV infection conditions.
